# The effect of pregnancy and puerperium on haemorrhage risk in women with cerebral cavernous malformations: a case-crossover study

**DOI:** 10.1093/esj/aakag130

**Published:** 2026-10-28

**Authors:** Matteo Palermo, Vittoria Viglione, Federico Tosto, Francesco Signorelli, Alessio Albanese, Alessandro Olivi, Francesco Doglietto, Marina Romozzi, Carmelo Lucio Sturiale

**Affiliations:** Department of Neurosurgery, Fondazione Policlinico Universitario Agostino Gemelli IRCCS, Università Cattolica del Sacro Cuore, Rome, Italy; Neurology Unit, Dipartimento di Neuroscienze, Organi di Senso e Torace, Fondazione Policlinico Universitario Agostino Gemelli IRCCS, Rome, Italy; Department of Neuroscience, “Giovanni Paolo II” Hospital, Lamezia Terme, Catanzaro, Italy; Department of Neurosurgery, Fondazione Policlinico Universitario Agostino Gemelli IRCCS, Università Cattolica del Sacro Cuore, Rome, Italy; Department of Neurosurgery, Fondazione Policlinico Universitario Agostino Gemelli IRCCS, Università Cattolica del Sacro Cuore, Rome, Italy; Department of Neurosurgery, Fondazione Policlinico Universitario Agostino Gemelli IRCCS, Università Cattolica del Sacro Cuore, Rome, Italy; Department of Neurosurgery, Fondazione Policlinico Universitario Agostino Gemelli IRCCS, Università Cattolica del Sacro Cuore, Rome, Italy; Neurology Unit, Dipartimento di Neuroscienze, Organi di Senso e Torace, Fondazione Policlinico Universitario Agostino Gemelli IRCCS, Rome, Italy; Department of Neurosurgery, Fondazione Policlinico Universitario Agostino Gemelli IRCCS, Università Cattolica del Sacro Cuore, Rome, Italy

**Keywords:** cavernoma, delivery, haemorrhage, pregnancy

## Abstract

**Introduction:**

Cerebral cavernous malformations (CCMs) are lesions that may be incidental or present with haemorrhage or focal neurological deficits. Some authors have reported apparent lesion enlargement during pregnancy and hypothesised that hormonal fluctuations may promote CCM growth or haemorrhage. Whether pregnancy increases the risk of symptomatic haemorrhage (SH) from CCMs therefore represents an important clinical concern.

**Patients and methods:**

We collected prospective data from patients with CCMs. A case-crossover framework was used to compare the risk of SH during pregnancy-related and non-pregnant observation periods. Pregnancy-related observation periods included pregnancy and the 6-week puerperium, while all other follow-up time after CCM diagnosis was considered non-pregnant. Each woman served as her own control, with the risk of SH during pregnancy-related periods compared with that during her non-pregnant follow-up periods.

**Results:**

After screening, the final cohort included 42 women diagnosed with a CCM at 46 years of age or younger. Of these, 23 women experienced at least 1 pregnancy (30.4% familial CCM; 21.7% brainstem CCM) at or after CCM diagnosis, accounting for 40 pregnancies. Overall, 2 SHs occurred during 25.6 pregnancy patient-years, corresponding to an annual SH rate of 7.8% (95% CI, 0.9–28.3), compared with 42 SHs during 420.7 non-pregnancy patient-years, corresponding to an annual SH rate of 10.0% (95% CI, 7.2–13.5). No statistically significant difference was found in the haemorrhagic rates between pregnant vs non-pregnant periods (*P* = .751). When observation periods were stratified according to a history of prior haemorrhage, no SHs occurred during 10.3 pregnancy patient-years among periods with prior haemorrhage, whereas 8 SHs occurred during 12.7 non-pregnancy patient-years, corresponding to an annual SH rate of 63.0% (95% CI, 27.2–124.2). Among periods without prior haemorrhage, 2 SHs occurred during 15.2 pregnancy patient-years, corresponding to an annual SH rate of 13.1% (95% CI, 1.6–47.5), compared with 34 SHs during 408.0 non-pregnancy patient-years, corresponding to an annual SH rate of 8.3% (95% CI, 5.8–11.6). In this subgroup, generalised estimating equation (GEE)-Poisson analysis showed no significant difference in haemorrhage risk between pregnancy and non-pregnancy periods (incidence rate ratio [IRR] 1.64, 95% CI, 0.44–6.13; *P* = .462). When analysis was restricted to brainstem CCMs, GEE-Poisson analysis showed no significant difference between pregnancy and non-pregnancy periods (IRR 2.09; 95% CI, 0.36–12.14; *P* = .411). One SH occurred during 8.2 pregnancy patient-years, corresponding to an annual SH rate of 12.2% (95% CI, 0.3–67.9), vs 6 SHs during 91.6 non-pregnancy patient-years, corresponding to 6.6% (95% CI, 2.4–14.3). Among periods with prior haemorrhage, no SHs occurred during 1.5 pregnancy patient-years (0.0%; 95% CI, 0.0–194.7), vs 2 SHs during 4.4 non-pregnancy patient-years (45.2%; 95% CI, 5.5–163.4). Among periods without prior haemorrhage, 1 SH occurred during 6.7 pregnancy patient-years (15.0%; 95% CI, 0.4–83.6), vs 4 SHs during 87.2 non-pregnancy patient-years (4.6%; 95% CI, 1.3–11.7), with no significant difference on GEE-Poisson analysis (IRR 3.50; 95% CI, 0.62–19.78; *P* = .156).

**Conclusion:**

Pregnancy was not associated with a significantly increased rate of SH in this cohort of women with CCMs. However, the limited number of events during pregnancy and the limited pregnant follow-up require cautious interpretation. Counselling should remain individualised according to lesion location, prior haemorrhagic history, neurological status and treatment history.

## Graphical abstract

**Graphical abstract:**
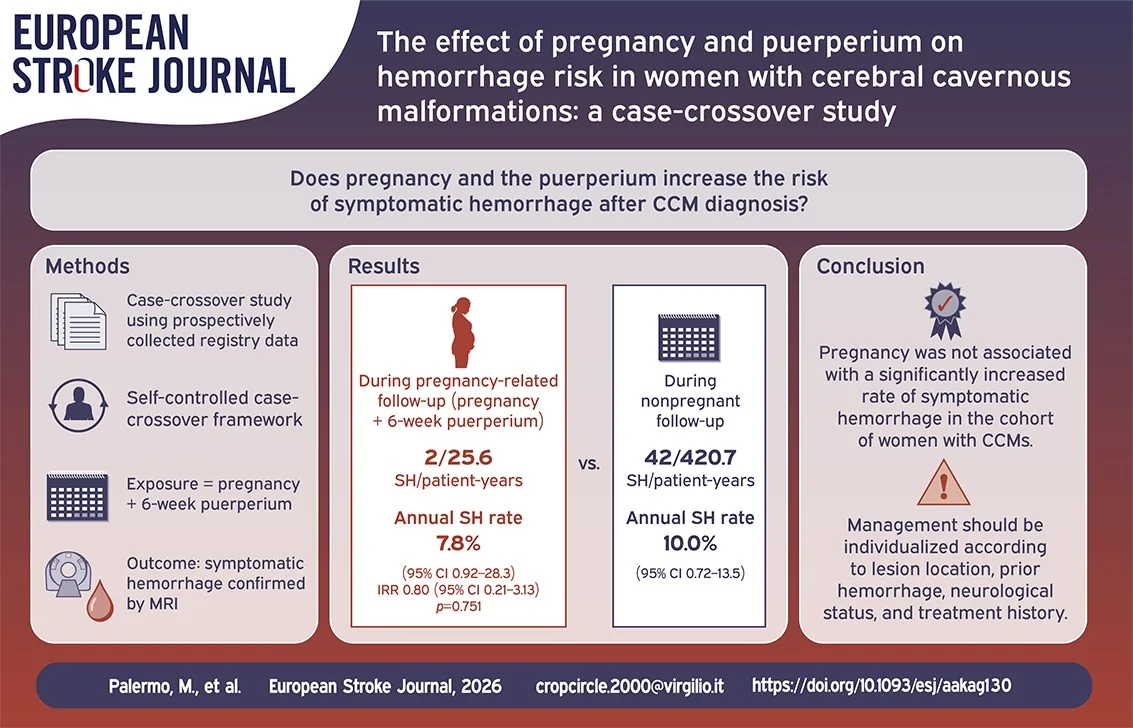

## Introduction

Cerebral cavernous malformations (CCMs) are vascular lesions composed of dilated, thin-walled sinusoidal channels with little or no intervening neural tissue.^1^ They may occur in sporadic or familial forms and are estimated to affect approximately 0.4%–0.8% of the general population.^2^ Although many CCMs remain asymptomatic and are discovered incidentally, symptomatic lesions may present with intracranial haemorrhage, focal neurological deficits, seizures or headache. In women of reproductive age, whether pregnancy increases the risk of CCM haemorrhage therefore represents an important clinical concern.^3^

Some authors have also reported apparent lesion enlargement during pregnancy and hypothesised that hormonal fluctuations, increased expression of vascular endothelial growth factor or basic fibroblast growth factor, pregnancy-related haemodynamic changes and endothelial proliferation may promote CCM growth or haemorrhage.^1–3^ However, evidence supporting these mechanisms remains limited.^1^

Early reports were sometimes interpreted as suggesting an increased haemorrhagic risk during pregnancy.^4^ More recent retrospective cohorts, however, have generally found comparable haemorrhage rates during pregnant and non-pregnant periods.^4–6^ These studies were limited by the assumption that each CCM had been present throughout the patient’s entire reproductive lifespan, including the years preceding radiological diagnosis. Because CCMs may develop de novo or become radiologically detectable over time, this approach may overestimate the duration of non-pregnant exposure and consequently underestimate the corresponding haemorrhage rate.^7^

Only 2 studies have prospectively evaluated haemorrhagic risk during pregnancy at or after CCM diagnosis, with apparently discordant findings. In their cohort of 64 patients restricted to brainstem CCMs, the annualised spontaneous haemorrhage rate was 20.4% during pregnancy compared with 7.2% during non-pregnant follow-up in the same patients.^2^ In contrast, Joseph et al. observed no haemorrhages during 32 prospectively followed pregnancies occurring after the diagnosis of a brain or spinal cord cavernous malformations (CMs), yielding an annual haemorrhage rate of 0.0% (95% CI, 0.0–13.6).^3^ Additionally, the authors found no statistically significant difference between pregnant and non-pregnant periods in the same patients.^3^ Yet, these studies provided limited characterisation of pregnancy-related comorbidities, pregestational haemorrhagic risk factors, obstetric events and postpartum risk modifiers.

The present study therefore aimed to determine whether pregnancy, delivery and the postpartum period are associated with an increased risk of symptomatic haemorrhage (SH) from CCMs.

## Patients and methods

### Study population and eligibility criteria

This was a case-crossover study based on prospectively collected data from patients with CMs. The study was conducted in accordance with the Declaration of Helsinki. The study adheres to the STROBE guidelines for case–control studies (Supplementary Material S2).

A case-crossover model was used to evaluate whether pregnancy is associated with an increased risk of symptomatic haemorrhage (SH).

Women of reproductive age (≤46 years) were included at the time of CCM diagnosis and were followed prospectively thereafter (Figure 1). For the present analysis, the prospective pregnancy group included women with at least one eligible pregnancy that was ongoing at the time of CCM diagnosis or occurred after diagnosis and before censoring. These women contributed pregnancy-related person-time during eligible pregnancy + puerperium intervals and non-pregnant person-time during all other eligible follow-up periods. Women with no eligible pregnancy ongoing at or after CCM diagnosis contributed exclusively non-pregnant person-time. Pregnancies completed before CCM diagnosis were not included in the primary exposure analysis but were recorded for descriptive purposes. When a woman experienced more than one eligible pregnancy, each pregnancy contributed a separate pregnancy-related exposure interval.

**Figure 1:**
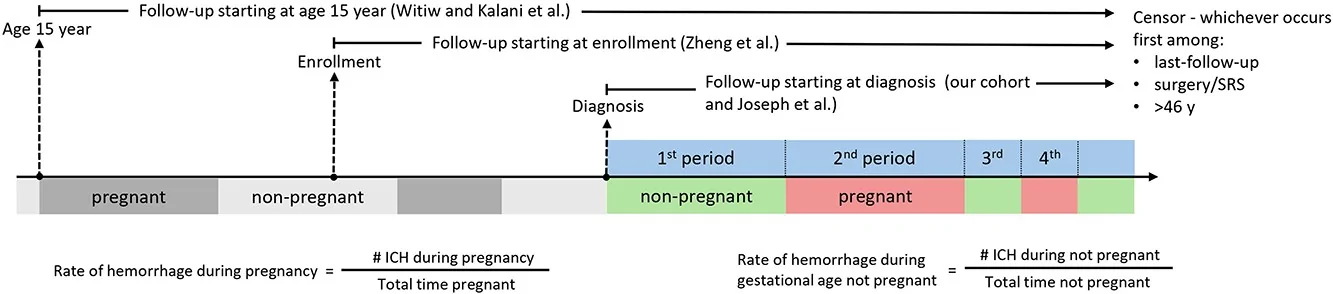
Schematic representation of follow-up definitions and person-period segmentation.

Patients were assumed to have the familial form if they had multiple lesions without a developmental venous anomaly (DVA).^3^ Patients were assumed to have the sporadic form of CM if they had a single lesion and a DVA or multiple lesions clustered around a DVA.^3^ In some instances, patients with multiple CCMs or suspected cerebral cavernomatosis underwent genetic testing to distinguish familial from sporadic disease, particularly when no other affected family members were known.

Exposure time was defined jointly as pregnancy + puerperium. Puerperium was considered as the subsequent 6 weeks postpartum or postabortion.^2^^,^^3^

In case of recurrence or residual, the patients were included in the analysis. In women who underwent surgery during pregnancy or within 6 weeks, follow-up was terminated at the date of surgery because they were considered disease-free after complete resection. In cases of partial resection, follow-up continued until the censoring date. Non-exposed time was defined as total post-enrolment follow-up minus the pregnancy-related exposure time (Figure 1).

Exclusion criteria were: (1) prior complete resection or radiotherapy with no residual or recurrent lesion on preconception MRI; (2) currently pregnant or breastfeeding at the time of the analysis; (3) concurrent vascular malformations or neoplasms; (4) missing data or lack of consent. After inclusion, all patients underwent prospective clinical follow-up. Spinal CMs were not excluded from the search strategy; however, no patients with spinal CMs were identified.

### Follow-up

All data were anonymised, study personnel received standardised training before initiation, and discrepancies in data reporting were resolved by site investigators. All patients were informed that they should notify the study team of any planned or unplanned pregnancy and/or any SHs after inclusion. To ensure adherence, scheduled yearly follow-up calls were made unless an in-person visit was scheduled. Pregnancy-related clinical information was abstracted from their medical records and pre- and post-inclusion medication change/addition/removal was documented; all imaging-confirming SHs were recorded. For pregnancies managed outside our institution, patients were contacted by telephone and asked to provide the relevant medical records covering the gestational period, including documentation of any pregnancy-related complications. For events managed at another institution, the corresponding clinical and imaging documentation was requested and reviewed at the subsequent follow-up visit to confirm whether the event met the predefined criteria for SH. Patients for whom adequate follow-up information could not be obtained were considered lost to follow-up.

Each patient provided at least 3 telephone numbers plus their home address and their email. Contact information was reviewed and updated at each visit. Magnetic resonance imaging was recommended at each follow-up whenever feasible and was mandatory at the onset of new neurologic symptoms. Follow-up was terminated at the time of treatment, death or age > 46 years, according to whichever occurred first. An age cutoff of 46 years was selected arbitrarily to define the reproductive-age period during which women remained at risk of pregnancy-related haemorrhage at or after CCM diagnosis and to guarantee comparability with the 2 previously published prospective studies (Figure 1).

### Endpoints

The primary outcome was specifically defined as SH: a haemorrhage associated with an acute or subacute onset of new or worsening functional neurological deficits confirmed by MRI from any CMs.^2^^,^^3^ Computed tomography findings alone were not considered sufficient for endpoint adjudication; when CT suggested haemorrhage, MRI confirmation of haemorrhage arising from the CCM was required for the event to be classified as SH. Accordingly, incidental or asymptomatic new haemorrhages were not considered. Symptomatic haemorrhages occurring at the time of CCM diagnosis, including those leading to the initial diagnosis, were considered as outcome events.

### Maternal and clinical variables

For each eligible pregnancy, maternal age at conception or during pregnancy, smoking status and relevant pre-existing comorbidities were recorded (Supplementary Material S1). Particular attention was given to conditions potentially associated with haemorrhagic risk, including chronic arterial hypertension and coagulation disorders present before pregnancy.

Radiologic variables included lesion location, maximal diameter on T1-weighted imaging and Zabramski type. In patients with multiple CCMs, the largest lesion was used to define lesion diameter. However, in the patients with multiple CMs and at least 1 brainstem CM, the brainstem lesion was considered as the index lesion regardless of whether it was the largest lesion. The timing of diagnosis in relation to pregnancy, and the clinical presentation at diagnosis were documented. Medication exposure was assessed both before and during pregnancy, including oral anticoagulants, vitamin K antagonists, prophylactic- or therapeutic-dose heparin, COX inhibitors, P2Y12 inhibitors, chronic non-steroidal anti-inflammatory drugs, chronic corticosteroids, selective serotonin reuptake inhibitors and antiepileptics.

Obstetric variables included pre-eclampsia, eclampsia, gestational hypertension, other relevant pregnancy complications, mode of delivery, gestational age at delivery and whether delivery was vaginal or caesarean. The management of the CCM during pregnancy, including conservative treatment, imaging surveillance and surgical or other interventions, was also recorded.

Postpartum variables included postpartum haemorrhage, need for blood transfusion, postpartum hypertension, postpartum depression, pulmonary embolism and other clinically relevant puerperal complications. Exposure to heparin, warfarin, aspirin, direct oral anticoagulants or other oral anticoagulant or antiplatelet therapies during the postpartum period was also documented.

Foetal and neonatal outcomes included congenital diseases or structural abnormalities, chromosomal abnormalities, preterm birth, foetal distress and other relevant perinatal complications.

### Statistical analysis

Descriptive statistics were used to summarise demographic, clinical and radiological characteristics. Continuous variables were reported as mean ± SD, whereas categorical variables were presented as frequencies and percentages. Comparisons between groups were performed using the Mann–Whitney *U* test for continuous variables and the chi-square test or Fisher’s exact test, as appropriate, for categorical variables.

Overall haemorrhage incidence was calculated as the number of SHs divided by the total person-years at risk. Pregnancy-associated incidence was calculated by dividing the number of SHs occurring during pregnancy or the puerperium by the corresponding person-time. The non-pregnant incidence was calculated using SHs and person-time accrued outside pregnancy and puerperium during reproductive-age follow-up (Figure 1). Incidence rates were expressed per patient-year, with 95% CIs derived from the Poisson distribution.

For the primary analysis, post-diagnosis follow-up was restructured into a person-period format. Each woman’s eligible follow-up was divided into consecutive periods according to pregnancy status. Post-diagnosis person-time occurring during pregnancy or the 6-week puerperium was classified as exposed, whereas all remaining eligible follow-up was classified as non-pregnant. For women diagnosed during pregnancy or puerperium, the diagnostic SH was assigned to the exposed period at the date of diagnosis, while only person-time from diagnosis onward contributed to the denominator. Women with more than 1 eligible pregnancy contributed a separate exposed period for each pregnancy. Women without an eligible pregnancy contributed only non-pregnant periods. Pregnancy-associated person-time was excluded from corresponding non-pregnant intervals to prevent double counting.

For each period, person-time was calculated in years. Age at the beginning of the period and the cumulative number of previous SHs were treated as time-varying covariates.

The association between pregnancy and SH was assessed using generalised estimating equation Poisson regression with a log link. An exchangeable working correlation structure and robust sandwich standard errors were used to account for the correlation among repeated periods contributed by the same patient. The logarithm of period-specific person-years was included as an offset. Non-pregnant periods were used as the reference category, and results were reported as incidence rate ratios (IRRs) with corresponding 95% CIs.

All tests were 2-sided, and a *P*-value < .05 was considered statistically significant. Statistical analyses were performed using R v.4.6.1. All analyses were reviewed by our institutional statisticians.

## Results

A total of 156 patients with CCM were screened. Of these, 108 were excluded: 56 were male, 12 had a history of prior treatment, 2 were pregnant or breastfeeding at enrolment, 3 had planned treatment, 3 had life-threatening, debilitating or severe medical conditions and 32 declined participation. All 12 patients had radiographically (MRI) confirmed obliteration. The remaining 48 patients were included. One patient was lost to follow-up, leaving 47 women with available follow-up data. Five women were diagnosed after 46 years of age and were excluded from the reproductive-age analysis. The final study cohort therefore included 42 women diagnosed with a CCM at 46 years of age or younger (Figure 2).

**Figure 2:**
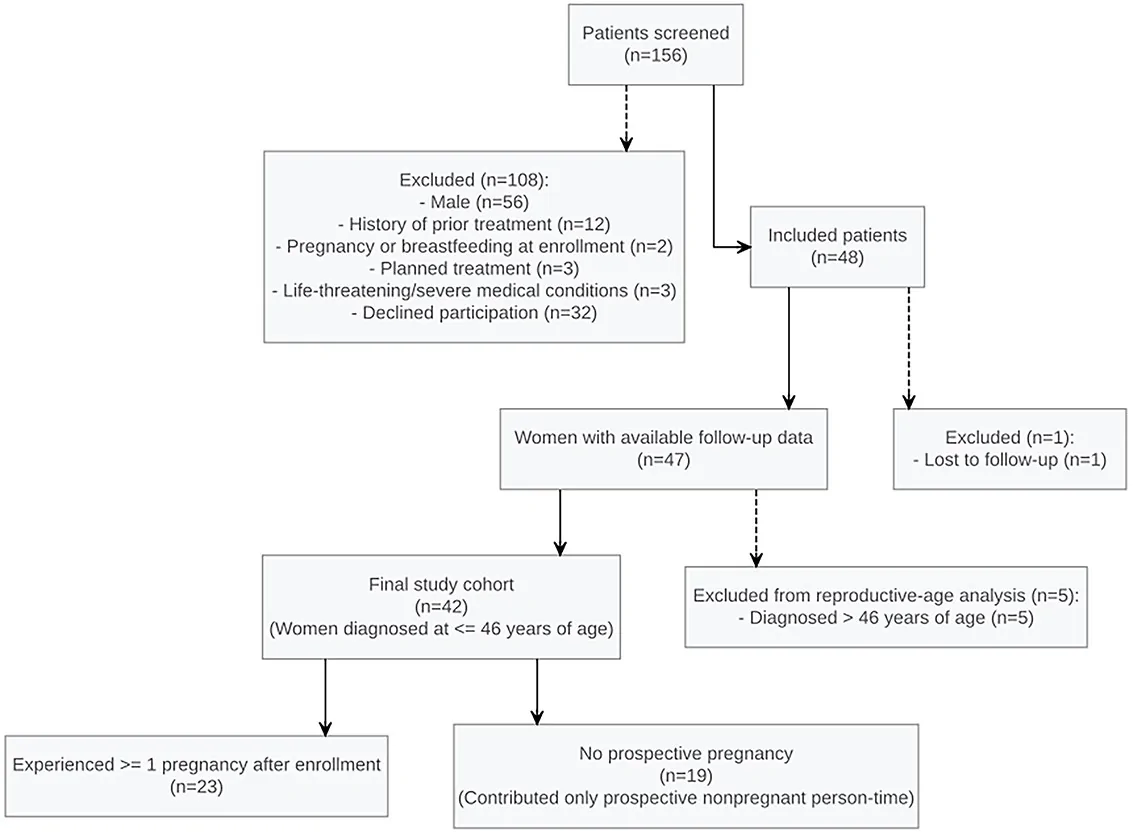
Flow diagram of patient selection.

### Study population and baseline characteristics

Among the 42 women included, 23/42 (54.8%) experienced at least 1 prospective pregnancy at or after CM diagnosis, whereas 19/42 (45.2%) had no prospective pregnancies (Table 1). Women with at least 1 prospective pregnancy were significantly younger at diagnosis than those without a prospective pregnancy (25.0 ± 7.7 vs 39.1 ± 4.1 years; *P* < .001). Among the 23 women with at least 1 prospective pregnancy, 40 pregnancies occurred at or after CM diagnosis, resulting in 35 live births and 5 non-viable pregnancies. Five additional pregnancies had occurred before CM diagnosis in this group, including 4 live births and 1 abortion.

**Table 1:** Demographic data of women diagnosed with CM at age 46 or younger.

		All women diagnosed at age 46 y or younger (*n* = 42)	With at least 1 prospective pregnancy (*n* = 23)	With no prospective pregnancy (*n* = 19)	*P*-value
**Age at diagnosis (mean years ± SD)**		31.4 ± 9.51	25.0 ± 7.7	39.1 ± 4.1	**<.001**
**Multiple lesions, %**		11 (26.2)	9 (39.1)	2 (10.5)	.075
**Familial form, %**		11 (26.2)	7 (30.4)	4 (21.1)	.726
**No. of patients presenting with clinical haemorrhage (brain or spine), %**		32 (76.2)	15 (65.2)	17 (89.5)	.083
**No. of patients presenting incidental finding, %**		10 (23.8)	8 (34.8)	2 (10.5)	
**Brainstem vs other location, %**		7 (16.7)	5 (21.7)	2 (10.5)	.428
**No. of patients with history of at least 1 pregnancy before CM diagnosis**		21 (50.0)	3 (13.0)	18 (94.7)^a^	**<.001**
**Total pregnancies**		83	45	38^b^	1.00
** Total live births**		71	39	32	.977
** Total non-viable foetal pregnancies**		12	6	6	
**Total prospective pregnancies**		40	40	–	–
** Total live births**		–	35	–	–
** Total non-viable foetal pregnancies**		–	5	–	–
**Total pregnancies before CM diagnosis^c^**		43	5	38^*^	**–**
**Total patients in which CM diagnosed at time of pregnancy or postpartum**		2	2	–	–
**Total clinical haemorrhage at diagnosis during pregnancy or postpartum**		2	2	–	–
**Total clinical haemorrhages during pregnancy after diagnosis (pregnant)**		0	0	–	–
**Total number of haemorrhages**		44	23	21	–
**Total number of haemorrhages at diagnosis**		32	15	17	–
**Total haemorrhage after diagnosis excluding those at diagnosis (non-pregnant)^c^**		12	8	4	–
**Total follow-up time after diagnosis to censor (not pregnant), y**		420.7	370.9	49.8	**–**
**Total follow-up time after diagnosis to censor (pregnant), y**		25.6	25.6	–	–

Abbreviation: CM = cavernous malformation.

a One patient did not have a pregnancy until after surgical resection of the cavernoma—exclusively contributing to non-gravid-person-years until resection.

b Three extra gestations occurred after surgical resection (after censoring)—not included.

c Patients diagnosed with CM during pregnancy did not have the index pregnancy included in this count.

Multiple lesions were identified in 9/23 women (39.1%) with a prospective pregnancy and in 2/19 women (10.5%) without a prospective pregnancy (*P* = .075). No significant between-group differences were observed in the frequency of familial CM (30.4% vs 21.1%; *P* = .726) or brainstem location (21.7% vs 10.5%; *P* = .428). Clinical haemorrhage was the presenting manifestation in 15/23 women (65.2%) with a prospective pregnancy and 17/19 women (89.5%) without a prospective pregnancy (*P* = .083); the remaining 8/23 women (34.8%) and 2/19 women (10.5%), respectively, were diagnosed incidentally. Twenty-one patients had experienced at least 1 pregnancy before CM diagnosis.

Among all women, only 2 were diagnosed with a CCM during pregnancy, and both presented with SH (Figure 3, pt. 6 and 19).

**Figure 3:**
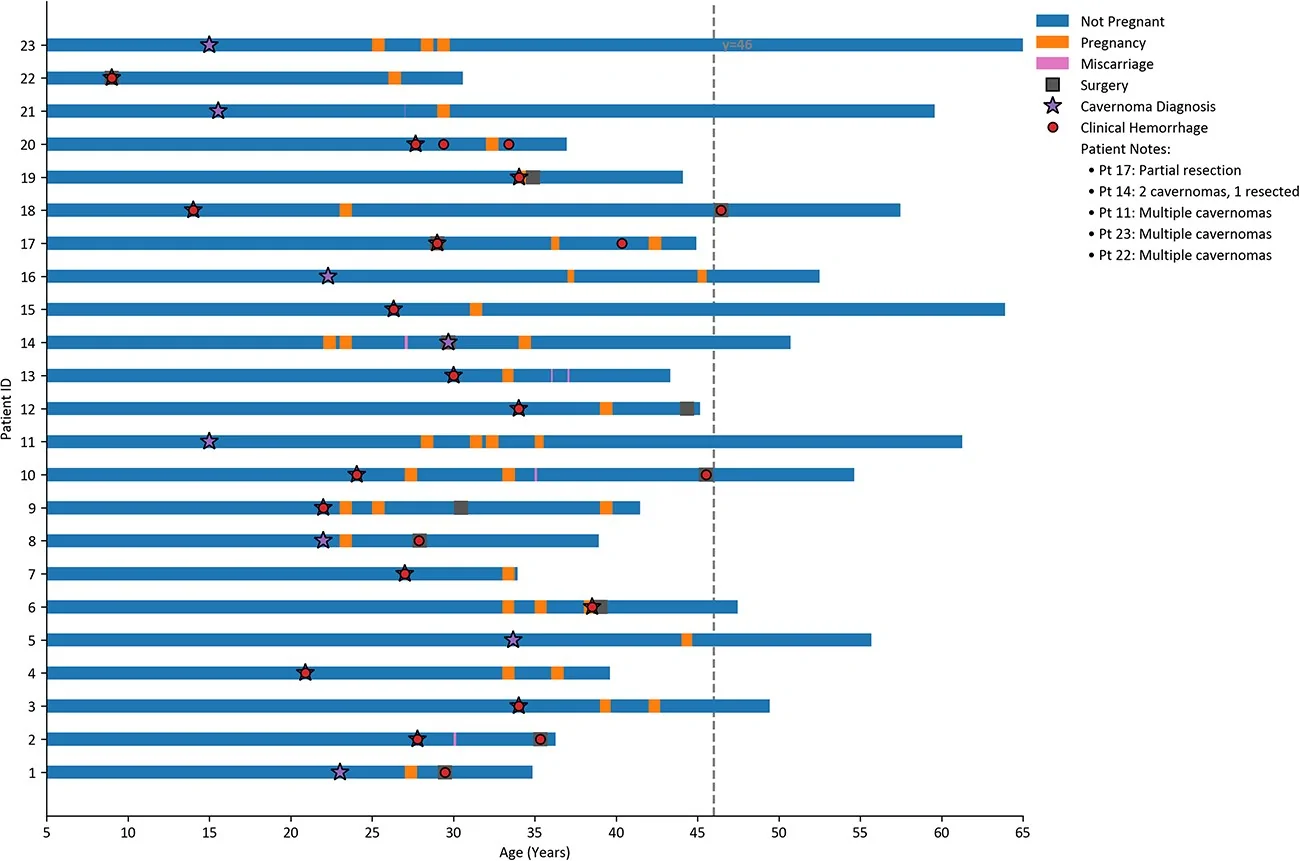
Individual patient timelines showing pregnancies, CCM diagnoses, clinical haemorrhages and surgical treatments of patients in the prospective pregnancy group. Abbreviation: CCM = cerebral cavernous malformation.

Patient 6 had experienced 2 previous uncomplicated pregnancies and was diagnosed during the third trimester of her third pregnancy after a bilateral tonic–clonic seizure associated with haemorrhage from a sporadic, left frontal Zabramski type II CM measuring 2 cm. She was treated with levetiracetam during pregnancy and delivered a live infant by caesarean section at 38 weeks. No postpartum complications occurred. Antiseizure therapy was subsequently changed to lacosamide 50 mg nightly, and the lesion was surgically resected 9 months after delivery. Instead, patient 19, aged 34 years, was diagnosed at 28 weeks of gestation following haemorrhage from a 4.5-cm pontine Zabramski type II CM. She delivered a live infant by caesarean section at 41 weeks. Her postpartum course was complicated by anaemia requiring blood transfusion, and the lesion was surgically treated 2 months after delivery. Neither patient had a history of miscarriage, and neither experienced any subsequent pregnancies after CCM resection.

### Symptomatic haemorrhage risk during pregnancy

Two SHs occurred during 25.6 pregnancy patient-years, corresponding to an annual SH rate of 7.8% (95% CI, 0.9–28.3). During non-pregnant follow-up, 42 SHs occurred over 420.7 patient-years, corresponding to an annual SH rate of 10.0% (95% CI, 7.2–13.5). The IRR from the generalised estimating equation (GEE)-Poisson model was 0.80 (*P* = .751; 95% CI, 0.21–3.13) (Table 2).

**Table 2:** Subgroup analysis of pregnancy-associated SH risk in 42 patients with 120 observation periods.

			Pregnancy period		Non-pregnancy period		GEE-Poisson (exchangeable), robust SE, IRR (95% CI)	*P*-value
Subgroup	No. of pts	No. of segs	No. of bleeding/ period, pt-y	Prospective ASHR, % 95% CI)	No. of bleeding/ period, pt-y	Prospective ASHR, % 95% CI)		
**Overall**	42	120	2/25.6	7.8 (0.9–28.3)	42/420.7	10.0 (7.2–13.5)	0.80 (0.21–3.13)	.751
Periods with prior SH								
**Yes**	19	21	0/10.3	0.0 (0.0–35.7)	8/12.7	63.0 (27.2–124.2)	NA^*^	NA^*^
**No**	39	99	2/15.2	13.1 (1.6–47.5)	34/408.0	8.3 (5.8–11.6)	1.64 (0.44–6.13)	.462
Periods with SH in previous year								
**Yes**	5	5	0/2.5	0.0 (0.0–122.1)	0/0.03	0.0 (0.0–1057.3)	NA^*^	NA^*^
**No**	42	115	2/23.1	8.7 (1.0–31.3)	42/420.4	10.0 (7.2–13.5)	0.90 (0.24–3.39)	.876

Abbreviations: ASHR = annual symptomatic haemorrhage rate; GEE = generalised estimating equation; IRR = incidence rate ratio; SH = symptomatic haemorrhage.

NA^*^: The GEE-Poisson models could not be estimated because no haemorrhagic events occurred during pregnancy.

Among observation periods without a prior haemorrhage, 2 SHs occurred during 15.2 pregnancy patient-years, corresponding to an annual SH rate of 13.1% (95% CI, 1.6–47.5). In comparison, 34 SHs occurred during 408.0 non-pregnant patient-years, corresponding to an annual SH rate of 8.3% (95% CI, 5.8–11.6). The GEE-Poisson model was 1.64 (*P* = .462; 95% CI, 0.44–6.13).

Among periods with a prior haemorrhage, no SHs occurred during 10.3 pregnancy patient-years, corresponding to an annual SH rate of 0.0% (95% CI, 0.0–35.7). Eight SH occurred during 12.7 non-pregnant patient-years, corresponding to an annual SH rate of 63.0% (95% CI, 27.2–124.2). The GEE-Poisson model could not be estimated because no haemorrhagic events occurred during pregnancy.

### Brainstem CMs

Among the 42 women included in the study, 7/42 (16.7%) had a brainstem CM (Table 3). Five (5/7) experienced at least 1 pregnancy ongoing at or after CM diagnosis, whereas 2/7 had no prospective pregnancies. Four patients had sporadic disease and 3/7 had a familial form; multiple CMs were present in 4/7 patients. Three (3/7) women presented with clinical haemorrhage, whereas 4/7 were diagnosed incidentally.

**Table 3:** Subgroup analysis of pregnancy-associated SH risk in 7 patients with 28 observation periods.

			Pregnancy period		Non-pregnancy period		GEE-Poisson (exchangeable), robust SE, IRR (95% CI)	*P*-value
Subgroup	No. of pts	No. of segs	No. of bleeding/ period, pt-y	Prospective ASHR, % 95% CI)	No. of bleeding/ period, pt-y	Prospective ASHR, % 95% CI)		
**Overall**	7	28	1/8.2	12.2 (0.3–67.9)	6/91.6	6.6 (2.4–14.3)	2.09 (0.36–12.14)	.411
**Periods with prior SH**								
**Yes**	3	4	0/1.5	0.0 (0.0–194.7)	2/4.4	45.2 (5.5–163.4)	NA^*^	NA^*^
**No**	7	24	1/6.7	15.0 (0.4–83.6)	4/87.2	4.6 (1.3–11.7)	3.50 (0.62–19.78)	.156
**Periods with SH in previous year**								
**Yes**	1	1	0/0.8	0.0 (0.0–389.3)	–	–	NA^*^	NA^*^
**No**	7	27	1/7.4	13.5 (0.3–75.0)	6/91.6	6.6 (2.4–14.3)	2.57 (0.60–11.0)	.202

Abbreviations: ASHR = annual symptomatic haemorrhage rate; GEE = generalised estimating equation; IRR = incidence rate ratio; SH = symptomatic haemorrhage.

NA^*^: The GEE-Poisson models could not be estimated because no haemorrhagic events occurred during pregnancy.

Overall, these 7 women experienced 13 pregnancies, including 8 vaginal and 5 caesarean deliveries. All pregnancies resulted in live births, and no foetal congenital anomalies were reported. Ten (10/13) pregnancies occurred at or after CM diagnosis: 7 resulted in vaginal delivery and 3 in caesarean delivery.

Other than patient 19, described above, no additional haemorrhagic events occurred during pregnancy. Overall, 1 SH occurred during 8.2 pregnancy patient-years, corresponding to an annual SH rate of 12.2% (95% CI, 0.3–67.9), compared with 6 SHs during 91.6 non-pregnancy patient-years, corresponding to an annual SH rate of 6.6% (95% CI, 2.4–14.3). GEE-Poisson analysis showed no significant difference in haemorrhage risk between pregnancy and non-pregnancy periods (IRR 2.09; 95% CI, 0.36–12.14; *P* = .411). When observation periods were stratified according to prior haemorrhage, no SHs occurred during 1.5 pregnancy patient-years among periods with a prior haemorrhage, corresponding to an annual SH rate of 0.0% (95% CI, 0.0–194.7), whereas 2 SHs occurred during 4.4 non-pregnancy patient-years, corresponding to an annual SH rate of 45.2% (95% CI, 5.5–163.4). The GEE-Poisson model could not be estimated in this subgroup because no events occurred during pregnancy. Among periods without a prior haemorrhage, one SH occurred during 6.7 pregnancy patient-years, corresponding to an annual SH rate of 15.0% (95% CI, 0.4–83.6), compared with 4 SHs during 87.2 non-pregnancy patient-years, corresponding to an annual SH rate of 4.6% (95% CI, 1.3–11.7). In this subgroup, GEE-Poisson analysis showed no significant difference between pregnancy and non-pregnancy periods (IRR 3.50; 95% CI, 0.62–19.78; *P* = .156).

## Discussion

In this cohort of women diagnosed with a CM during reproductive age, pregnancy was not associated with a significantly increased rate of SHs compared with non-pregnant follow-up. The annual SH rate was 7.8% (95% CI, 0.9–28.3) during pregnancy-related periods and 10.0% (95% CI, 7.2–13.5) during non-pregnant periods.

Our findings are consistent with the earlier cohorts of Witiw et al.^8^ and Kalani and Zabramski.^6^ The former reported 3 haemorrhages during 261.4 pregnancy patient-years and 46 during 4519 non-pregnant patient-years, corresponding to rates of 1.15% and 1.01% per patient-year.^8^ Kalani and Zabramski observed 5 SHs among 168 pregnancies and concluded that pregnancy did not increase haemorrhage risk in either sporadic or familial CM.^6^ However, both studies were based on retrospective reproductive histories and assumed that the CM had been present throughout the women’s reproductive years, which may underestimate the true rate since CMs can arise de novo,^7^ while also introducing recall bias. Our study differs from previous studies in that it assessed the prospective risk of haemorrhage only during pregnancies occurring at or after CM diagnosis: this avoids assumptions about whether the lesion had been present throughout the woman’s reproductive years before its definitive identification.

Our results are similar to those of Joseph et al., who evaluated haemorrhage risk beginning at CM diagnosis rather than assuming lifelong lesion presence.^3^ In their cohort, 4 women experienced haemorrhage during pregnancy or postpartum, and each event led to the initial CM diagnosis. No haemorrhage occurred during 32 subsequent pregnancies after CM diagnosis (26.9 pregnancy patient-years), vs 42 haemorrhages occurring during 402.6 non-pregnant patient-years. In our study, the 2 pregnancy-associated SHs also represented diagnostic events, with no subsequent haemorrhage during pregnancy after diagnosis. In those with a known diagnosis of CM, no haemorrhages occurred during subsequent pregnancies, whereas the risk of haemorrhage while non-pregnant was 10.0% per year. In a review of multiple case reports and series of patients from our group,^1^ we found that in just over half of cases reported with detail, the haemorrhage led to the first diagnosis of CM, and only very few cases of haemorrhage from CM are documented to have occurred in those with known CM diagnosis. The very low incidence of prospective haemorrhage may partly reflect self-selection, as women who experienced haemorrhage during a previous pregnancy may have been less likely to pursue subsequent pregnancies. Indeed, in both of our cases, surgery was performed shortly after delivery, and neither woman subsequently experienced another pregnancy. In the study by Joseph et al.,^3^ all 4 women who presented with SH from a newly diagnosed CM during pregnancy subsequently had additional pregnancies without further haemorrhage, as did all 4 women reported by Kalani and Zabramski.^6^

Within our brainstem CM subgroup of 7/42 patients (16.7%), 5 women experienced at least one pregnancy ongoing at or after CM diagnosis. Three women presented with clinical haemorrhage, whereas 4 were diagnosed incidentally. One pregnancy-associated SH occurred, corresponding to an annual SH rate of 12.2% (95% CI, 0.3–67.9) during pregnancy-periods compared with 6.6% (95% CI, 2.4–14.3) during non-pregnancy patient-years. GEE-Poisson analysis showed no significant difference in haemorrhage risk between pregnancy and non-pregnancy periods (IRR 2.09; 95% CI, 0.36–12.14; *P* = .411). Although the point estimate was numerically higher during pregnancy, the small number of patients and events in our cohort precludes firm conclusions regarding pregnancy-associated haemorrhagic risk in this subgroup.

These findings should be interpreted within the broader literature. Earlier studies generally did not identify pregnancy as a major determinant of CM haemorrhage risk,^6^^,^^8^ whereas the recent prospective multicentre study by Zheng et al., restricted to women with brainstem CMs, reported a higher annual SH rate during pregnancy-related periods than during non-pregnant periods (20.4% vs 7.2%; adjusted IRR 2.56).^2^ Differences in case mix may partly account for the apparent discrepancy. Unlike our anatomically heterogeneous cohort, in which brainstem CMs represented only 16.7% of the sample, Zheng et al. exclusively enrolled women with brainstem CMs, and included a cohort highly enriched for previous haemorrhagic presentation.^2^

Moreover, Zheng et al. found that SH within the preceding year was associated with pregnancy-related SH, with older maternal age also emerging as a potential risk factor.^2^ This observation suggests that the timing of the most recent haemorrhage may be particularly relevant for preconception risk stratification. Our cohort was too small to assess this potential risk modifier and therefore cannot confirm or refute this association.

### Implications for clinical practice

From a practical standpoint, the available evidence supports an individualised approach to preconception counselling and pregnancy management in women with CMs. The interval between haemorrhage and conception may be particularly relevant in patients with a recent SH. Bektas et al. suggested that conception within the first year after haemorrhage may increase the risk of rebleeding and complicate perinatal management,^9^ whereas earlier studies proposed waiting up to 2.5 years after lesion stabilisation before attempting pregnancy.^6^^,^^8^ More recent evidence has similarly suggested that a recent SH may be more relevant to pregnancy-related haemorrhagic risk than a remote history of haemorrhage alone.^2^ However, evidence supporting a specific waiting interval remains limited, and any potential benefit of postponing conception should be balanced against maternal age and individual reproductive considerations.^2^ Accordingly, particularly in women with a recent SH or a brainstem CM, conception may reasonably be deferred until clinical and radiological stability has been achieved, although the optimal duration of such stability remains uncertain.

The optimal mode of delivery for these patients remains unclear, requiring further investigation in larger prospective registries. In our cohort, SHs occurred predominantly during the second and third trimesters, with no events during labour or delivery, in line with previous observations.^2^^,^^3^ Nevertheless, the limited number of events prevents conclusions regarding trimester-specific risk or whether the mode of delivery modifies haemorrhagic risk. Caesarean delivery has sometimes been favoured because of concerns regarding haemodynamic changes, Valsalva manoeuvres and transient increases in intracranial pressure during vaginal delivery.^10^^,^^11^ However, given the low-flow nature of CMs and the lack of convincing evidence that vaginal delivery increases haemorrhagic risk, the mode of delivery should generally be determined by obstetric indications, maternal neurological status and individualised multidisciplinary assessment.^10^^,^^11^

Management of an SH occurring during pregnancy should likewise be individualised according to haemorrhage severity, neurological status, lesion location and gestational age. When the maternal neurological condition is stable, conservative management may allow pregnancy to continue and promote further foetal maturation.^2^^,^^3^ Conversely, neurological deterioration, recurrent haemorrhage or clinically significant mass effect may require neurosurgical intervention during pregnancy, with delivery subsequently planned according to maternal and foetal conditions.^3^ Although severe pregnancy-associated SH appears uncommon, its potential consequences for both mother and foetus justify close clinical surveillance.

In these circumstances, close multidisciplinary collaboration among neurosurgeons, obstetricians, anaesthesiologists and neonatologists is essential. Cavernous malformation resection may need to be performed while allowing the pregnancy to continue, followed by delivery once an acceptable gestational age has been reached.^3^ In clinical practice, modifiable factors should be addressed proactively, including careful blood pressure management and avoidance of potentially relevant behavioural or pharmacological risk factors, although the extent to which these measures prevent CM haemorrhage remains uncertain.

### Limitations and strengths

This study has several strengths. Pregnancy and non-pregnancy were treated as time-varying exposure periods, and the person-period structure allowed individual women to contribute observation time to both states. Additionally, our study initiated prospective follow-up at inclusion and collected detailed, individual obstetric timelines. Outcome definitions and analytic approaches also differed. We required both clinical deterioration and MRI evidence to define SH, whereas some earlier series relied primarily on symptoms.^6^^,^^8^

Follow-up was censored at clinically relevant endpoints, and the analysis avoided attributing the entire reproductive lifespan before CM diagnosis to an existing lesion. The study also included detailed clinical, radiological, and obstetric information and evaluated a mixed-location cohort that may be more representative of general CM practice than a population restricted to brainstem lesions.

Several limitations must also be acknowledged. First, the small cohort and the occurrence of only 2 pregnancy-associated SHs resulted in wide CIs and limited statistical power. Second, our study was characterised by a high proportion of haemorrhagic presentations (32/42, 76.2%), likely reflecting referral and severity-selection bias related to recruitment at a tertiary centre. Consequently, our study population may represent women with more symptomatic or higher-risk CCMs, limiting the generalisability of our findings, particularly to patients with incidentally discovered or asymptomatic lesions. One possibility to explain the very low haemorrhage rate during pregnancy is that a proportion of patients who became pregnant once and subsequently received a CM diagnosis or suffered a haemorrhage may have opted not to pursue further pregnancies. Another potential confounder is that patients with sporadic CM were censored after surgery, and the decision to pursue intervention may have been influenced by the potentially higher risk for rehaemorrhage. Stratification according to prior SH resulted in small subgroups and wide CIs, limiting the precision of these estimates. Therefore, subgroup findings should be interpreted as exploratory. The low event count also prevented reliable multivariable adjustment and estimation of the effect of prior haemorrhage. The small number of brainstem lesions precluded sufficiently powered location-specific analyses and limits direct comparison with the high-risk population studied by Zheng et al.^2^

Additionally, our annualised haemorrhage rate during non-pregnant follow-up (10.0% per patient-years) was higher than that reported in the largest prospective population-based study of CCMs, which found a 5-year risk of 2.4% for first intracranial haemorrhage and 29.5% for recurrent haemorrhage.^12^ However, direct comparison is limited by differences in outcome definition and cohort composition, as our estimate included both first and recurrent haemorrhagic events and was restricted to women of reproductive age. Finally, obstetric outcomes and delivery modes could not be compared with an unaffected control group.

## Conclusion

Pregnancy was not associated with a significantly increased rate of SH in this cohort of women with CCMs. However, the low number of events during pregnancy and the limited pregnant follow-up require cautious interpretation. Counselling should remain individualised according to lesion location, prior haemorrhagic history, neurological status and treatment history.

## Supplementary Material

Supplementary_material_aakag130

## Data Availability

Available upon reasonable request by e-mailing the corresponding author.
