# Supplementary material for: The effect of pregnancy and puerperium on haemorrhage risk in women with cerebral cavernous malformations: a case-crossover study
Source: Eur Stroke J. 2026 Oct 28;11(10):aakag130. doi: 10.1093/esj/aakag130 (PMC13714789; doi:10.1093/esj/aakag130)
Supplement: Supplementary file 1 [file supplementary_material_aakag130.zip › Supplementary_material.docx]

**SUPPLEMENTARY**

**Table S1:** Pregnancy Data of Women Diagnosed With CM at Age 46 or Younger.

**Table S2:** Strobe reporting checklist for case-control studies.
