# Supplementary material for: The effect of pregnancy and puerperium on haemorrhage risk in women with cerebral cavernous malformations: a case-crossover study
Source: Eur Stroke J. 2026 Oct 28;11(10):aakag130. doi: 10.1093/esj/aakag130 (PMC13714789; doi:10.1093/esj/aakag130)
Supplement: Supplementary file 1 [file supplementary_material_aakag130.zip › Tables S1-S2.docx]

**S1.** Pregnancy Data of Women Diagnosed With CM at Age 46 or Younger.

|  | | | **Prospective patients - retrospective pregnancies (n=3/23, pregn.=5)** | **Prospective patient – prospective pregnancies (n=23, pregn.=40)** | **Non-prospective patients –retrospective pregnancies (n=18, pregn.=38)** |
| --- | --- | --- | --- | --- | --- |
| Age at pregnancy (mean years ± SD) | | | 28.0 ± 5.8 | 32.8 ± 5.9 | 31.4 ± 6.4 |
| Pre-gestation comorbidities | | |  |  |  |
|  | Hypertension | | 0 | 11 | 6 |
|  | Chronic steroid use | | 0 | 2 | 2 |
|  | Chronic NSAID use | | 0 | 4 | 3 |
|  | SSRI | | 0 | 0 | 6 |
|  | Any bleeding disorder (Raynaud, V Laiden, MTHFR) | | 0 | 3 | 4 |
|  | Aspirin | | 0 | 0 | 4 |
|  | LMWH | | 0 | 0 | 1 |
|  | HMWH | | 0 | 0 | 0 |
|  | P2Y12 | | 0 | 0 | 0 |
|  | DOAC | | 0 | 0 | 0 |
|  | VKA | | 0 | 0 | 0 |
|  | Antiseizure medication | | 0 | CBZ (2);  LCM + LEV (1);  LTG (1); LEV (4) | 0 |
| Gestational complications | | |  |  |  |
|  | Eclampsia | | 0 | 1 | 0 |
|  | Preeclampsia | | 0 | 3 | 0 |
|  | Fetal congenital syndromes | | 0 | 4 (ASD, ARPKD, 2*) | 2 (DS, EA) |
|  | ARDS | | 0 | 3 | 1 |
|  | Mode of delivery | |  |  |  |
|  |  | Vaginal | 2 | 18 | 19 |
|  |  | Cesarean | 2 | 17 | 13 |
| Post-partum comorbidities | | |  |  |  |
|  | Hypertension | | 3 | 6 | 0 |
|  | Aspirin | | 0 | 0 | 4 |
|  | LMWH | | 0 | 0 | 3 |
|  | HMWH | | 0 | 0 | 0 |
|  | DOAC | | 0 | 0 | 0 |
|  | VKA | | 0 | 0 | 0 |
|  | Postpartum Hemorrhage | | 1 | 5 | 2 |
|  | Transfusion | | 0 | 3 | 3 |
|  | Anemia | | 2 | 3 | 1 |
|  | Post-partum depression | | 0 | 5 | 1 |
|  | PE | | 0 | 1 | 0 |
| EA: Esophageal atresia, DS: Down syndrome; ASD: autism-spectrum disorder; ARPKD: autosomal recessive polycystic kidney disease; *: the authors cannot disclose the status the other two patients; NSAID: non-steroidal anti-inflammatory medication; VKA: vitamin K antagonist; LMWH: low-molecular weight heparin; HMWH: high-molecular weight heparin; ARDS: acute-respiratory distress syndrome; DOAC: direct-oral anticoagulant; CBZ: Carbamazepine; LCM: Lacosamide; LEV: Levetiracetam; LTG: Lamotrigine | | | | | |

**Results**

*Pregnancy characteristics and outcomes*

Overall, 86 pregnancies in 42 women were recorded. Three pregnancies occurred after surgical resection and censoring and were therefore excluded from the prospective follow-up analysis. The remaining 83 pregnancies included 71/83 (85.5%) live births and 12/83 (14.5%) nonviable pregnancies.

The mean maternal age at the beginning of pregnancies occurring after diagnosis was 32.8 ± 5.9 years. Among pregnancies that had occurred before the CCM was diagnosed, the mean maternal age at pregnancy onset was 28.0 ± 5.8 years in women who later experienced at least one prospective pregnancy and 31.4 ± 6.4 years in women who had no pregnancies ongoing at or after CM diagnosis (**S1)**

Among the 23 women with at least one prospective pregnancy, 40 pregnancies occurred at or after CM diagnosis, resulting in 35 live births and 5 nonviable pregnancies. Five additional pregnancies had occurred before CM diagnosis in this group, including 4 live births and 1 abortion. Among women who experienced the 40 prospective pregnancies, a total of 23 SHs were recorded over the entire observation period: 15/23 (65.2%) at CCM diagnosis and 8 (34.8%) during subsequent non-pregnant follow-up segments. Women who presented with SH at CCM diagnosis subsequently experienced 22 pregnancies before censoring, none of which complicated by recurrent SH. Among the 40 prospective pregnancies, 18/40 (45.0%) resulted in vaginal delivery, 17 (42.5%) in cesarean delivery, and 5 (12.5%) ended in abortion. Pre-gestational conditions or treatments included hypertension in 11/40 (27.5%) pregnancies, chronic corticosteroid use in 2/40 (5.0%), chronic nonsteroidal anti-inflammatory drug use in 4/40 (10.0%), bleeding disorders in 3/40 (7.5%), and treatment with antiseizure medications in 8/40 (20.0%). Gestational complications included 1/40 case of eclampsia (2.5%), 3 cases of preeclampsia (7.5%), 4 fetal congenital syndromes (10.0%), and 3 cases of acute respiratory distress syndrome (7.5%). Among patients receiving antiseizure medications, only patient 12 (**Figure 3**) treated with carbamazepine had a child diagnosed with autism spectrum disorder, while all the other fetuses developed without complications. Post-gestational events included hypertension in 6/40 (15.0%) pregnancies, postpartum hemorrhage in 5/40 (12.5%), transfusion in 3/40 (7.5%), anemia in 3/40 (7.5%), postpartum depression in 5/40 (12.5%), and pulmonary embolism in 1/40 (2.5%).

Among the 18 women with pregnancies before diagnosis who did not subsequently experience a prospective pregnancy, 38 retrospective pregnancies were recorded, resulting in 32/38 (84.2%) live births and 6 (15.8%) abortions. One additional woman classified as having no prospective pregnancy did not become pregnant until after surgical resection and censoring. Seventeen of 19 women (89.5%) presented with SH at CCM diagnosis. Overall, 21 SHs were recorded: 17 (81.0%) at diagnosis and 4 (19.0%) during subsequent prospective nonpregnant follow-up.

**S2.** STROBE Statement—checklist of items that should be included in reports of observational studies

|  | Item No. | Recommendation | Page  No. | Relevant text from manuscript |
| --- | --- | --- | --- | --- |
| **Title and abstract** | 1 | (*a*) Indicate the study’s design with a commonly used term in the title or the abstract | 1 | The effect of pregnancy… |
|  |  | (*b*) Provide in the abstract an informative and balanced summary of what was done and what was found | 1 | The effect of pregnancy… |
| Introduction | | | |  |
| Background/rationale | 2 | Explain the scientific background and rationale for the investigation being reported | 4 | Cerebral cavernous malformations (CCMs) are angiographically… |
| Objectives | 3 | State specific objectives, including any prespecified hypotheses | 4 | Cerebral cavernous malformations (CCMs) are angiographically… |
| Methods | | | |  |
| Study design | 4 | Present key elements of study design early in the paper | 5-8 | This was a retrospective cohort… |
| Setting | 5 | Describe the setting, locations, and relevant dates, including periods of recruitment, exposure, follow-up, and data collection | 5-8 | This was a retrospective cohort… |
| Participants | 6 | (*a*) *Cohort study*—Give the eligibility criteria, and the sources and methods of selection of participants. Describe methods of follow-up  *Case-control study*—Give the eligibility criteria, and the sources and methods of case ascertainment and control selection. Give the rationale for the choice of cases and controls  *Cross-sectional study*—Give the eligibility criteria, and the sources and methods of selection of participants | 5-8 | This was a retrospective cohort… |
|  |  | (*b*) *Cohort study*—For matched studies, give matching criteria and number of exposed and unexposed  *Case-control study*—For matched studies, give matching criteria and the number of controls per case | 5-8 | This was a retrospective cohort… |
| Variables | 7 | Clearly define all outcomes, exposures, predictors, potential confounders, and effect modifiers. Give diagnostic criteria, if applicable | 5-8 | This was a retrospective cohort… |
| Data sources/ measurement | 8* | For each variable of interest, give sources of data and details of methods of assessment (measurement). Describe comparability of assessment methods if there is more than one group | 5-8 | This was a retrospective cohort… |
| Bias | 9 | Describe any efforts to address potential sources of bias | 5-8 | This was a retrospective cohort… |
| Study size | 10 | Explain how the study size was arrived at | 5-8 | This was a retrospective cohort… |

Continued on next page

| Quantitative variables | 11 | Explain how quantitative variables were handled in the analyses. If applicable, describe which groupings were chosen and why | 5-8 | This was a retrospective cohort… |
| --- | --- | --- | --- | --- |
| Statistical methods | 12 | (*a*) Describe all statistical methods, including those used to control for confounding | 5-8 | This was a retrospective cohort… |
|  |  | (*b*) Describe any methods used to examine subgroups and interactions | 5-8 | This was a retrospective cohort… |
|  |  | (*c*) Explain how missing data were addressed | 5-8 | This was a retrospective cohort… |
|  |  | (*d*) *Cohort study*—If applicable, explain how loss to follow-up was addressed  *Case-control study*—If applicable, explain how matching of cases and controls was addressed  *Cross-sectional study*—If applicable, describe analytical methods taking account of sampling strategy | 5-8 | This was a retrospective cohort… |
|  |  | (*e*) Describe any sensitivity analyses | 5-8 | This was a retrospective cohort… |
| Results | | | | |
| Participants | 13* | (a) Report numbers of individuals at each stage of study—eg numbers potentially eligible, examined for eligibility, confirmed eligible, included in the study, completing follow-up, and analysed | 8-11 | A total of 156 patients were screened. Of these, 52… |
|  |  | (b) Give reasons for non-participation at each stage | 8-11 | A total of 156 patients were screened. Of these, 52… |
|  |  | (c) Consider use of a flow diagram | 8-11 | A total of 156 patients were screened. Of these, 52… |
| Descriptive data | 14* | (a) Give characteristics of study participants (eg demographic, clinical, social) and information on exposures and potential confounders | 8-11 | A total of 156 patients were screened. Of these, 52… |
|  |  | (b) Indicate number of participants with missing data for each variable of interest | 8-11 | A total of 156 patients were screened. Of these, 52… |
|  |  | (c) *Cohort study*—Summarise follow-up time (eg, average and total amount) | 8-11 | A total of 156 patients were screened. Of these, 52… |
| Outcome data | 15* | *Cohort study*—Report numbers of outcome events or summary measures over time | 8-11 | A total of 156 patients were screened. Of these, 52… |
|  |  | *Case-control study—*Report numbers in each exposure category, or summary measures of exposure | 8-11 | A total of 156 patients were screened. Of these, 52… |
|  |  | *Cross-sectional study—*Report numbers of outcome events or summary measures | 8-11 | A total of 156 patients were screened. Of these, 52… |
| Main results | 16 | (*a*) Give unadjusted estimates and, if applicable, confounder-adjusted estimates and their precision (eg, 95% confidence interval). Make clear which confounders were adjusted for and why they were included | 8-11 | A total of 156 patients were screened. Of these, 52… |
|  |  | (*b*) Report category boundaries when continuous variables were categorized | 8-11 | A total of 156 patients were screened. Of these, 52… |
|  |  | (*c*) If relevant, consider translating estimates of relative risk into absolute risk for a meaningful time period | 8-11 | A total of 156 patients were screened. Of these, 52… |

Continued on next page

| Other analyses | 17 | Report other analyses done—eg analyses of subgroups and interactions, and sensitivity analyses | 8-11 | A total of 156 patients were screened. Of these, 52… |
| --- | --- | --- | --- | --- |
| Discussion | | | | |
| Key results | 18 | Summarise key results with reference to study objectives | 11-14 | In this cohort of women diagnosed with a CM during… |
| Limitations | 19 | Discuss limitations of the study, taking into account sources of potential bias or imprecision. Discuss both direction and magnitude of any potential bias | 11-14 | In this cohort of women diagnosed with a CM during… |
| Interpretation | 20 | Give a cautious overall interpretation of results considering objectives, limitations, multiplicity of analyses, results from similar studies, and other relevant evidence | 11-14 | In this cohort of women diagnosed with a CM during… |
| Generalisability | 21 | Discuss the generalisability (external validity) of the study results | 11-14 | In this cohort of women diagnosed with a CM during… |
| Other information | |  | | |
| Funding | 22 | Give the source of funding and the role of the funders for the present study and, if applicable, for the original study on which the present article is based | 2 | Funding: Not applicable |

*Give information separately for cases and controls in case-control studies and, if applicable, for exposed and unexposed groups in cohort and cross-sectional studies.

**Note:** An Explanation and Elaboration article discusses each checklist item and gives methodological background and published examples of transparent reporting. The STROBE checklist is best used in conjunction with this article (freely available on the Web sites of PLoS Medicine at http://www.plosmedicine.org/, Annals of Internal Medicine at http://www.annals.org/, and Epidemiology at http://www.epidem.com/). Information on the STROBE Initiative is available at www.strobe-statement.org.
